# The Evolution of Nutrient and Microbial Composition and Maturity During the Composting of Different Plant-Derived Wastes

**DOI:** 10.3390/biology14030268

**Published:** 2025-03-06

**Authors:** Yuxin Xie, Pengbing Wu, Ying Qu, Xingchi Guo, Junyan Zheng, Yuhe Xing, Xu Zhang, Qian Liu

**Affiliations:** 1 College of Garden, Changchun University, Changchun 130012, China; xieyuxin0401@163.com (Y.X.); ww202309_cc@163.com (P.W.); 18104311229@163.com (Y.Q.); cc2024_09cc@163.com (X.G.); luckzhang0111@163.com (J.Z.); jdyyluck@sina.com (Y.X.); yl2023_zz@163.com (X.Z.); 2 Institute of Resource Utilization and Soil Conservation, Changchun University, Changchun 130022, China

**Keywords:** plant-derived waste, composting, nutrient, humification, microbial community, phytotoxicity, resource utilization

## Abstract

Composting is an environmentally friendly treatment technology that recycles and sanitizes organic solid waste. This study aimed to assess the evolution of nutrients, maturity, and microbial communities during the composting of different plant-derived wastes. The composting process was conducted over 49 days using three types of plant-derived waste: wheat bran (WB), peanut straw (PS), and poplar leaf litter (PL). This process was examined through physical, chemical, and biological parameters. The results revealed that after 49 days of composting, the three groups experienced significant changes. They were odorless, were insect-free, exhibited a dark brown color, had an alkaline pH value, and had an electrical conductivity (EC) value of less than 4 mS/cm. These characteristics indicated that they had reached maturity. Nutrient content was the most significant factor influencing the degree of humification of the different composting materials, while changes in microbial community diversity were the key driving factors. Significantly, the compost PS, derived from peanut straw, entered the thermophilic phase first, and by the end of composting, it had the lowest organic matter (OM) loss rate (17.4%), with increases in total nitrogen (TN), total phosphorus (TP), and total potassium (TK) in the order of PS > PL > WB. The increase in humus carbon (HSC) content and the humic acid/fulvic acid (HA/FA) ratio followed the order PS > WB > PL. FTIR spectra indicated that PS had greater aromatic characteristics compared to the other samples. The abundance and diversity of bacterial and fungal communities in the compost increased significantly, accompanied by more complex community structures. Crucially, there were no phytotoxic effects in any of the three composting treatments, and the compost PS boasted a high germination index (GI) of 94.79%, with the lowest heavy metal contents. The findings indicate that the compost PS has the highest potential for resource utilization and is suitable for agricultural applications. Our results demonstrate that composting technology for plant-derived waste has the potential to enhance soil fertility and provide a reference for the composting treatment and resource utilization of other plant-derived waste.

## Simple Summary

Composting plant-derived waste into nutrient-rich fertilizers offers a sustainable solution for organic waste management and soil enhancement. This study evaluated the composting efficiency of three plant-derived materials—wheat bran (WB), peanut straw (PS), and poplar leaf litter (PL)—over 49 days, focusing on nutrient dynamics, microbial community shifts, and compost maturity. The results showed that all composts achieved maturity, characterized by stable pH, low electrical conductivity, and the absence of phytotoxicity. Notably, PS compost exhibited superior performance: it entered the thermophilic phase earliest, retained the highest nutrient levels (total nitrogen, phosphorus, and potassium), and displayed the greatest humification (highest humus content and humic acid/fulvic acid ratio). Microbial diversity in PS was richer, with metabolic functions driving decomposition. FTIR analysis confirmed stronger aromaticity in PS, linked to advanced humification. Crucially, PS compost demonstrated the highest germination index (94.79%) and low heavy metal contents, confirming its agricultural safety. These findings highlight peanut straw as the most promising feedstock for composting, combining efficient nutrient retention, robust microbial activity, and high-quality maturity. This study provides actionable insights for optimizing plant-derived waste composting, supporting sustainable agriculture and circular economy practices.

## 1. Introduction

Plant-derived waste, including crop residues, horticultural waste, and forestry by-products, is prevalent in agricultural and horticultural production and has long been regarded as low-value waste [1]. However, recent advancements in sustainable agriculture have highlighted their potential as renewable resources, particularly through composting—a process that converts these organic materials into nutrient-rich fertilizers. Such fertilizers not only improve soil fertility and reduce dependence on synthetic alternatives but also align with global priorities for environmental conservation and circular economy practices [2]. Given the urgent need to address resource efficiency and ecological sustainability, optimizing the valorization of plant-derived waste through composting has become a critical research focus.

Composting relies on microbial-driven biodegradation, where nutrient dynamics are governed by shifts in microbial composition and the progression of compost maturity [3]. Key factors influencing this process include feedstock characteristics (e.g., carbon-to-nitrogen ratio, lignin content) and environmental parameters such as temperature, aeration, and moisture levels [4,5]. Variations in feedstock composition directly impact microbial activity, thereby affecting decomposition rates and the quality of the final product [6,7]. Additionally, the species and abundance of microorganisms in the compost environment change significantly during the process of degradation. Therefore, the evolution of microbial communities during composting is a key factor in determining the maturity and stability of compost [8]. The dynamic changes in the structure and function of microbial communities affect the decomposition rate and eventual stability of organic matter from the early stage of aerobic decomposition to the middle stage of anaerobic degradation, and finally to the late stage of stability [9,10]. Different plant-derived wastes may provide varied conditions for different microbial species, influencing the composting process and final product quality [11].

Compost maturity serves as a key indicator of product quality, reflecting the extent of organic matter stabilization and pathogen reduction [12]. The degree to which compost materials are transformed into stable organic matter is commonly referred to as maturity [13]. According to different composting conditions, the compost decomposition time required for waste from different sources is different. Based on previous studies of most plant-derived waste (such as corn stalks, mixtures of wheat and castor bran, and leaves), the compost maturation cycle is usually 30–60 days [14,15,16]. High-maturity compost products have fewer pathogenic microorganisms and higher soil improvement functions [17]. The differences in the compost maturity evolution of different plant-derived wastes may be related to the amount of complex organic matter in raw materials, which includes cellulose and lignin, as well as the degree of microbial activity [18,19]. Humus generation is critically influenced by both physicochemical characteristics and the structural dynamics of microbial communities [20]. The decomposition of plant-derived waste generates heat and other substances that could be harmful to plant growth [21]. Therefore, whether the compost product is fully mature is very important. Indicators of full maturity include the appearance, color, and odor, temperature, carbon nitrogen ratio, changes in organic matter content, humification index, and seed germination index [22]. A previous study found that the porosity, carbon and nitrogen content, and other physical and chemical properties of agricultural and forestry wastes with different high-carbon sources can also significantly change the microbial succession and functional characteristics of aerobic compost, thus affecting the degree of maturity [23]. However, it is still unclear how the microbial community structure of different plant-derived wastes affects the quality of compost products. The mechanism of this process needs to be further studied. Consequently, understanding the changes in the maturity of various plant-derived wastes during the composting process is crucial for evaluating the effectiveness of compost products in agricultural applications.

Furthermore, comparative studies on the evolution of nutrient and microbial composition during the composting of plant-derived waste are not comprehensive. To deeply understand the differences in the maturity and stability of different plant-derived wastes in the composting process, it is essential to investigate the dynamic differences in material transformation and microbial community composition during the composting process of these materials. This would provide scientific bases for the optimization of the composting process and the production of organic fertilizer.

In this study, the selected plant-derived waste research objects represent crop residues (wheat bran, peanut straw) and forestry by-products (poplar leaf litter), which cover the typical types of plant-derived waste in agroforestry and horticultural ecosystems. In addition, the carbon/nitrogen (C/N) ratio of the three raw materials showed a significant gradient difference: wheat bran was a high-nitrogen raw material, while peanut straw and poplar litter were medium- and high-carbon source materials, respectively [24]. This difference can provide an important reference for the composting of other waste. And these three compost materials are the main food crops and keynote tree species in northern China, especially in Jilin Province. Their waste can increase significantly, especially in October. Traditional incineration, landfilling, and other treatment methods easily cause resource waste and improper chemical circulation, which is also an urgent problem to be solved [25]. Therefore, we proposed two hypotheses: (1) Peanut straw, as a composting raw material, may exhibit higher composting efficiency, richer microbial diversity, and a stronger degree of humification than wheat bran and poplar leaf litter. (2) All three treatments have good agricultural safety, which solves the environmental impact and agricultural problems of plant-derived waste.

Current studies on nutrient transformation and microbial dynamics during composting lack comprehensive comparisons across diverse plant-based feedstocks. To address this gap, this study aims to (1) analyze physicochemical variations in compost derived from distinct plant residues; (2) characterize microbial community succession using high-throughput sequencing; (3) identify key drivers of humification through Partial Least Squares Path Modeling (PLS-PM); and (4) assess the maturity levels of compost products for agricultural applicability.

## 2. Materials and Methods

### 2.1. Composting Materials and Trials

#### 2.1.1. Composting Materials and Experimental Design

The wheat bran and peanut straw used as raw materials for composting were purchased from Chenyang Grass Limited Company (Hebei, China). Poplar leaf litter (including leaf litter of *Populus alba* var. *pyramidalis Bge.*, *Populus alba × P. Berolinensis*, and *Populus pseudosimonii Kitag.*) was directly collected from the Changchun University West campus (125°17’56″ E, 43°49′47″ N), street (125°19’26″ E, 43°54′16″ N), residential area (125°20′25″ E, 43°50′20″ W), and Park (125°18′48″ E, 43°51′55″ W). After collection, it was coarsely crushed.

**Figure 1:**
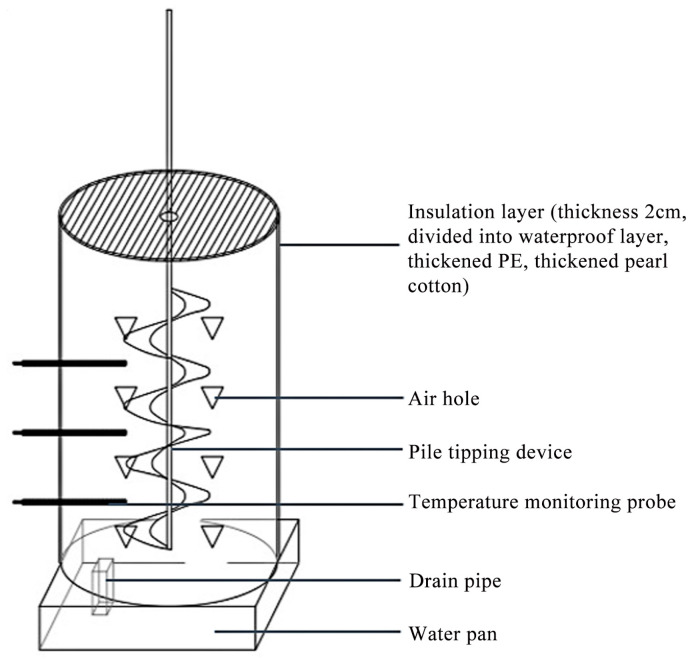
Schematic diagram of composting device.

Based on the organic matter content of three plant-derived waste types, urea supplementation was utilized to modulate the carbon-to-nitrogen (C/N) ratio of the composting feedstock. The C/N ratio was optimized to approximately 30:1, with moisture content maintained at 65–70%. The treatments, designated as WB, PS, and PL, were replicated thrice (Table 1). The composting experiment was conducted in a custom-built reactor (50 cm diameter × 80 cm height; Figure 1) over 49 days. During the thermophilic and heating phases, the piles were turned every 48 h, while the cooling phase required turning intervals of 72 h. The experiment was carried out at the experimental teaching base of Changchun University at the end of September 2023.

#### 2.1.2. Sampling Protocol

Temperature monitoring probes were vertically installed at three positions (top, center, and bottom) of the reactor for daily temperature recording at 10:00 AM. Systematic sampling was conducted across five decomposition phases: initial, peak thermophilic, thermophilic, cooling, and curing stages. Each treatment group included quintuplicate samples collected according to the schedule detailed in Table 2.

#### 2.1.3. Sample Processing Methodology

Tripartite 400 g specimens from different reactor levels were homogenized using the quartering technique. Fresh subsamples were immediately analyzed for pH and electrical conductivity (EC). Parallel aliquots underwent two preservation protocols: refrigeration at 4 °C and ambient air-drying. Processed samples were pulverized through 0.25 mm sieves for subsequent characterization of organic constituents (the determination of OM, TN, TP, TK) and humic substances (humic carbon (HSC), humic acid carbon (HAC), fulminate carbon (FAC)), complemented by infrared spectroscopic analysis.

#### 2.1.4. Microbiological Preservation Strategy

Additional 200 g specimens collected during three critical phases (initial, peak thermophilic, and curing) were cryopreserved at −80 °C for microbial community analysis. All experimental procedures incorporated triplicate biological replicates to ensure statistical reliability.

**Figure 2:**
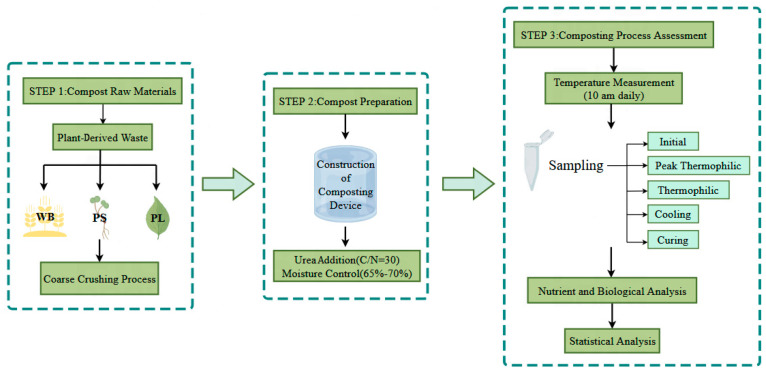
The flow-sheet of the composting treatment.

The flow-sheet of the experiment is represented in Figure 2.

### 2.2. Analytical Methods

#### 2.2.1. Bioassay and Analytical Procedures

The germination index (GI) of Chinese cabbage seeds was measured using the 1:10 (*w*/*v*) water extract bioassay to assess the phytotoxicity of compost, following the method previously described by Feng et al. [26]. Physicochemical parameters were characterized using standardized instrumentation: pH values were recorded with a calibrated digital pH meter, and electrolyte profiles were determined by conductivity meter measurements.

#### 2.2.2. Nutrient Element Analysis

Organic matter content was quantified via potassium dichromate external heating oxidation [27]. Nitrogen speciation employed Kjeldahl distillation methodology [28]. Phosphorus levels were established through molybdenum–antimony (Sb) colorimetric detection [29]. Potassium quantification utilized flame photometric analysis with appropriate calibration standards [30].

#### 2.2.3. Humic Substance Characterization

Humus carbon fractions were analyzed following International Humic Substances Society protocols [31]. Sequential extraction techniques isolated humic, humic acid, and fulvic acid components, with organic carbon content determined through dichromate oxidation.

The infrared spectra of HA and FA were determined by iS10 Fourier transform infrared spectrometer (Thermo Fisher Scientific, Waltham, MA, USA). The structural evolution of humic fractions was investigated using lyophilized specimens analyzed by FT-IR spectroscopy (4000–500 cm^−1^ spectral range), focusing on functional group transitions [32].

#### 2.2.4. Microbial Community Profiling

The DNA extraction and high-throughput sequencing of cryopreserved compost samples were performed by Biomarker Technologies Limited Company (Beijing, China). Sequence analysis and microbial community profiling were also conducted by the company. Microbial diversity was analyzed using the Illumina NovaSeq 6000 sequencing platform (Biomarker Technologies, China, Beijing), employing 16S rRNA V3-V4 sequencing for bacteria and ITS sequencing for fungi. The original image data files obtained by high-throughput sequencing were converted into Sequenced Reads by Base Calling analysis. The results were stored in FASTQ (fq) file format, which contains the sequence information of Reads and their corresponding sequencing quality information. The raw sequence data have been deposited in the National Center for Biotechnology Information Sequence Read Archive (BioProject ID: PRJNA1227704; study accession number: SRP566070). The temporal sampling strategy and experimental replicates are systematically presented in Table 3.

### 2.3. Data Processing

Initial dataset curation and preprocessing were conducted using Microsoft Excel 365 (v16.83) for tabular organization and descriptive statistics. Parametric analyses involving one-way ANOVA with post hoc Duncan tests (*p* = 0.05 significance threshold) were executed in SPSS Statistics 27.0 (IBM Corp., Armonk, New York, NY, USA). Multivariate statistical visualizations encompassing Pearson correlation matrices and functional annotation plots were developed using OriginPro 2021 (9.8.5.157). Beta diversity patterns were evaluated through nonmetric multidimensional scaling (NMDS) implemented in R 4.3.0 (R Core Team, Vienna, Austria), employing the vegan (v2.6-4) and plspm (v0.5.1) packages for ecological distance computation and Partial Least Squares Path Modeling (PLS-PM), respectively. For microbial analysis, the functional analysis of bacterial communities relies on the FAPROTAX functional prediction platform. That of fungal communities relies on the Fungi Functional Guild (FUNGuild), a tool that can be used for the taxonomic analysis of fungi by ecological societies. The Alpha diversity index of the sample was evaluated using QIIME2 2020.6 software. All graphical outputs were optimized for publication standards using vector-based editing protocols.

## 3. Results and Discussion

### 3.1. Basic Physical and Chemical Indices in Composting Process

#### 3.1.1. Temperature

**Figure 3:**
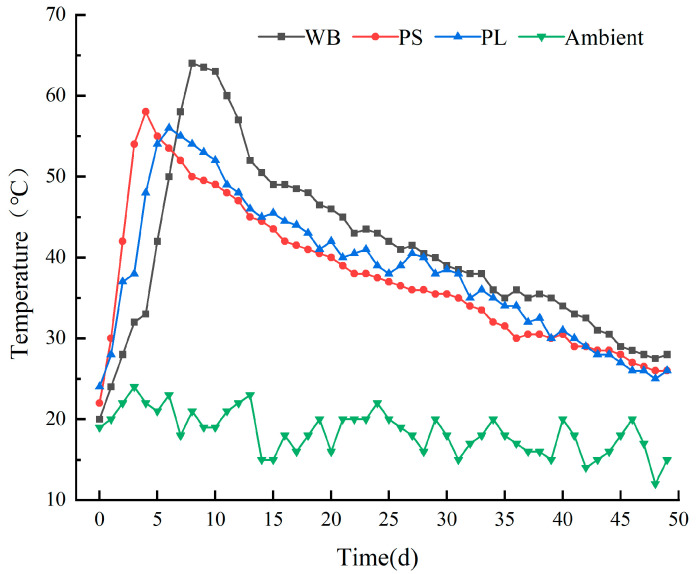
Temperature changes during the three compost treatments.

Changes in the composting temperature for each treatment are shown in Figure 3. Based on the temperature variations, the composting process can be divided into four stages: the initial stage, thermophilic stage, cooling stage, and maturation stage. WB, PS, and PL entered the thermophilic stage on days 8, 4, and 6, respectively, with peak temperatures reaching 64 °C, 58 °C, and 56 °C. All treatments maintained temperatures above 50 °C for 6–8 days, meeting the thermal requirements for compost maturation. The sustained high-temperature environment effectively aids in the elimination of pathogenic microorganisms [33,34]. Among the three treatments, PS entered the thermophilic stage the fastest, while WB achieved the highest temperature. These variations could be mechanistically associated with feedstock-specific architectural properties and dynamic microbiome reconfiguration during biostabilization processes [35]. As reported by El-mrini et al., the thermophilic phase exhibited biostimulatory effects, where exothermic metabolic cascades from the intensified microbial mineralization of labile organics generated self-sustaining thermal feedback [36,37].

#### 3.1.2. pH and EC Values

**Figure 4:**
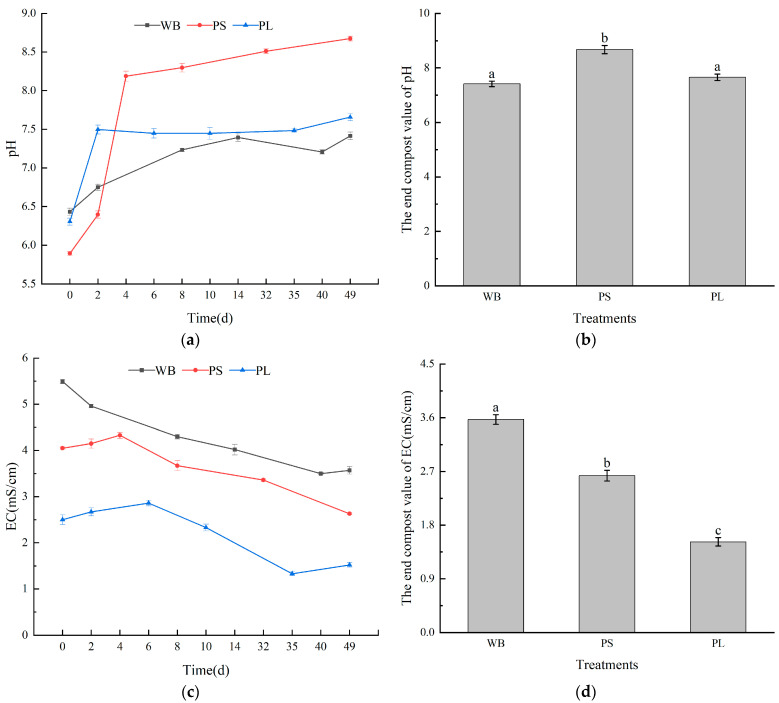
Changes in pH and EC values in the three compost treatments: (**a**) the change in the pH value during the three treatments of composting, (**b**) the change in the pH value at the end of the three treatments of composting, (**c**) the change in the EC value during the three treatments of composting, and (**d**) the change in the EC value at the end of composting. Different letters represent significant differences between different composting treatments (*p* < 0.05).

pH serves as a crucial biogeochemical regulator in composting systems, mediating microbial metabolic shifts that govern organic mineralization and nutrient retention [38]. In particular, the pH value of the peanut straw (PS) treatment increased significantly, which may have facilitated higher pH levels under high-temperature conditions, thus accelerating decomposition (Figure 4a) [39]. Concomitant with thermogenic progression, all experimental groups demonstrated pH trajectories shifting from neutral to alkaline regimes (final pH: 7–9; Figure 4b), indicative of microbially mediated deacidification processes during organic matrix mineralization.

The EC value is an important indicator of the soluble salt concentration in a compost extract [40]. Excessive soluble salts in compost can be toxic to plants, hindering their growth. The changes in EC values in the treatment of three kinds of plant-derived waste are shown in Figure 4c. The initial EC values of WB, PS, and PL were 5.49 mS/cm, 4.05 mS/cm, and 2.50 mS/cm, respectively (Figure 4d). Wheat bran contains a relatively high soluble salt content; when it is used as a composting material, the soluble salt content is reduced, transforming the waste into organic fertilizer. After composting, the EC value of all three piles was below 4 mS/cm, meeting composting standards and indicating that the composts were nearly mature [41]. In addition, the EC values of both the PS and PL treatments increased when the temperature increased and decreased when the temperature decreased. This was because during the thermophilic period, there was greater microbial activity in the reactor, which promoted the conversion of organic matter into various mineral salts, resulting in an increase in the EC value. Conversely, after the composts entered the cooling stage, the decrease in reactor temperature led to the generation of nitrate nitrogen, which in turn caused a decrease in the EC value [42].

### 3.2. Nutrient Content in the Composting Process

**Figure 5:**
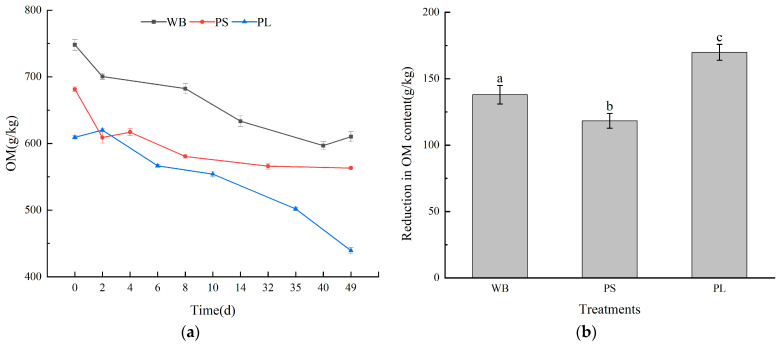
Changes in organic matter in the three treatments: (**a**) changes in organic matter in the composting process of the three treatments and (**b**) reductions in organic matter content in the three treatments. Different letters represent significant differences between different composting treatments (*p* < 0.05).

The biodegradation kinetics in aerobic composting systems are predominantly driven by carbonaceous substrate utilization, where heterotrophic microbial consortia mediate sequential C-source transformation through enzymatic cascade reactions. As evidenced by Figure 5, differential organic matter (OM) depletion patterns emerged across treatments, exhibiting substrate-specific mineralization coefficients (WB: 137.93 g/kg; PS: 118.31 g/kg; PL: 169.85 g/kg) after 49-day biostabilization. Notably, the PS matrix demonstrated attenuated OM mineralization (17.3% mass reduction) compared to the other two treatments, a phenomenon mechanistically analogous to the cation exchange modulation observed in attapulgite-enhanced sludge composting systems [43]. This suppressed carbon flux suggests the presence of PS-specific recalcitrant fractions (potentially suberin polyphenolic complexes) requiring extended humification pathways.

Among the three treatments, the C/N ratios in the treatments of WB, PS, and PL raw materials were 14.89, 25.26, and 88.09, respectively. After composting and decomposing, the final C/N ratios dropped to 10.58, 11.28, and 17.80. They all met the standard of aerobic compost decomposition to achieve harmlessness [44]. In addition, the carbon and nitrogen of poplar deciduous waste itself were relatively high, but the carbon content treated with PL after maturation was better degraded, which is consistent with Xu’s research results [45].

**Figure 6:**
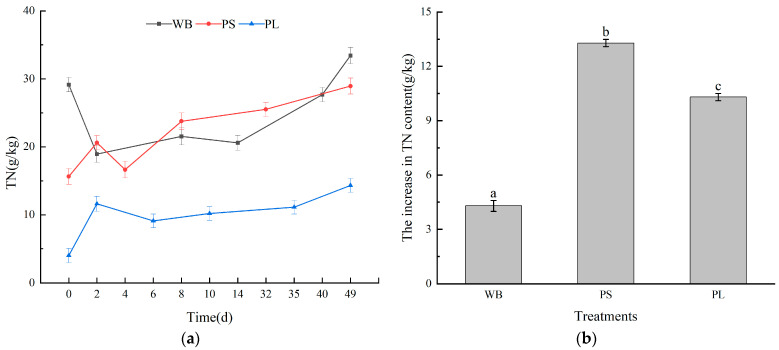

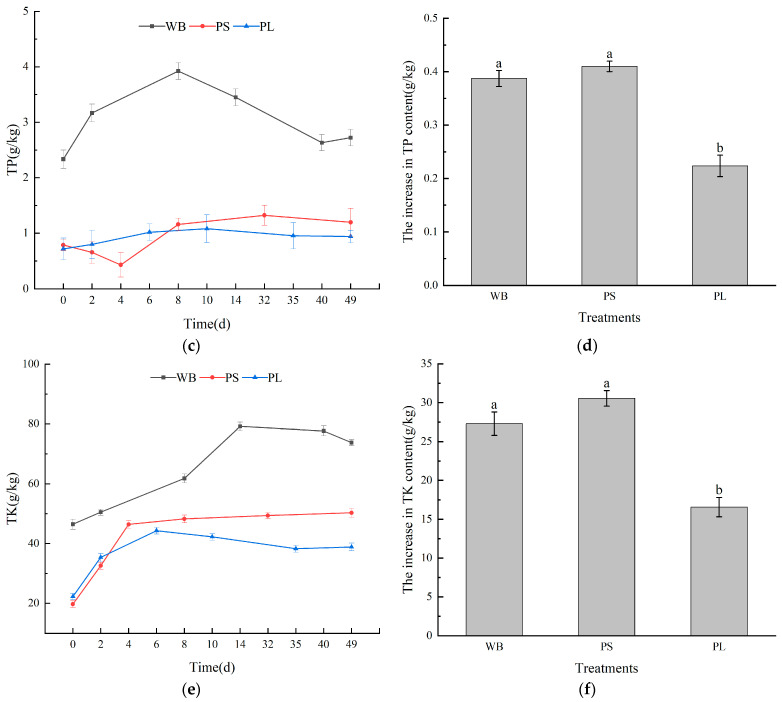
Changes in nutrient content in the three treatments: (**a**) changes in TN content in the three treatments, (**b**) increases in TN in the three treatments, (**c**) changes in TP content in the three treatments, (**d**) increases in TP in the three treatments, (**e**) changes in TK content in the three treatments, and (**f**) increases in TK in the three treatments. Different letters represent significant differences between different composting treatments (*p* < 0.05).

During the composting process, carbon sources serve as the primary energy source for microorganisms, while nitrogen sources play a key role in regulating biosynthesis processes [46]. Divergent nitrogen transformation patterns emerged among lignocellulosic feedstocks during bioconversion (Figure 6a,b). Initial TN concentrations diverged substantially across substrates (WB: 29.12 ± 0.45 g/kg; PS: 15.64 ± 0.28 g/kg; PL: 4.01 ± 0.11 g/kg). Post-treatment analyses revealed differential enrichment gradients: WB maintained nitrogen conservation superiority (ΔTN +14.7%, final 33.40 g/kg), and PS demonstrated maximum bioaccumulation efficiency (ΔTN +84.9%, final 28.90 g/kg), while PL exhibited constrained assimilation (ΔTN +71.9%, final 6.89 g/kg). This hierarchy suggests feedstock-specific mineralization–immobilization turnover mechanisms, where WB’s dense lignocellulose matrix likely limited ammonification losses, whereas PL’s labile carbon architecture facilitated enhanced nitrous emissions through denitrification pathways.

Our investigation revealed differential nitrogen accumulation patterns among plant-derived waste materials throughout the 49-day composting cycle. Initial nitrogen quantification demonstrated substantial variability across treatments: wheat bran (WB) contained 29.12 g/kg, peanut shells (PS) 15.64 g/kg, and plant litter (PL) 4.01 g/kg (Figure 6b). Post-composting analysis showed nitrogen enrichment rates of 14.7% (WB), 84.9% (PS), and 71.9% (PL), respectively. Notably, WB maintained superior nitrogen retention capacity, achieving the highest final nitrogen concentration among treatments. PS exhibited the most pronounced nitrogen accumulation rate, suggesting efficient nitrogen conservation mechanisms during its decomposition. Conversely, PL demonstrated a relatively limited nitrogen gain, indicative of substantial nitrogen depletion during leaf matter degradation. These differential outcomes likely stem from complex nitrogen transformation processes involving competing pathways of microbial immobilization and mineralization [47]. Thermophilic phase analysis revealed treatment-specific nitrogen dynamics: PS displayed significant nitrogen accretion, whereas PL showed marginal increases. This thermal-stage divergence suggests varying magnitudes of ammoniacal nitrogen volatilization across substrates, particularly pronounced in leaf-based composts. The observed nitrogen losses in PL systems underscore the need for targeted mitigation strategies in lignocellulosic waste composting, particularly during high-temperature phases [48].

In the process of composting and decomposing plant-derived waste, phosphorus was not easily volatilized, so the total phosphorus (TP) content did not change significantly. As shown in Figure 6c,d, at the end of composting, the TP content of the three kinds of plant-derived waste increased by 16.6%, 52.0%, and 31.2%, respectively, and the TP content of the PS pile increased the most. According to reports, in the process of aerobic composting, microorganisms decomposed organic matter at the same time, releasing volatile gases such as ammonia. The mass and volume of the pile were also reduced, and the loss of water led to a “concentration effect” [49]. This led to a significant increase in the three treatments during the thermophilic phase of composting, which also indicated that a large amount of phosphorus participates in the conversion of substances at this stage, and the composting effect was better. As shown in Figure 6e,f, the total potassium (TK) content of each composting process increased, and this amount of potassium was not lost in the form of volatilization during the composting process. The TK content of WB, PS, and PL increased by 36.9%, 60.7%, and 42.6%, respectively, after 49 days of composting, and the TK content of PS showed the largest increase, while the TK content of WB was higher than that of the other two composts. The above-mentioned effect made the content of TP, TK, and other nutrients in the compost increase after the aerobic compost was decomposed, making the compost a nutrient-rich organic fertilizer. In addition, the content of TP and TK in the PS treatment increased more after composting, which may be because the “concentration effect” of the peanut straw pile was the strongest.

### 3.3. Humus Carbon in the Composting Process

**Figure 7:**
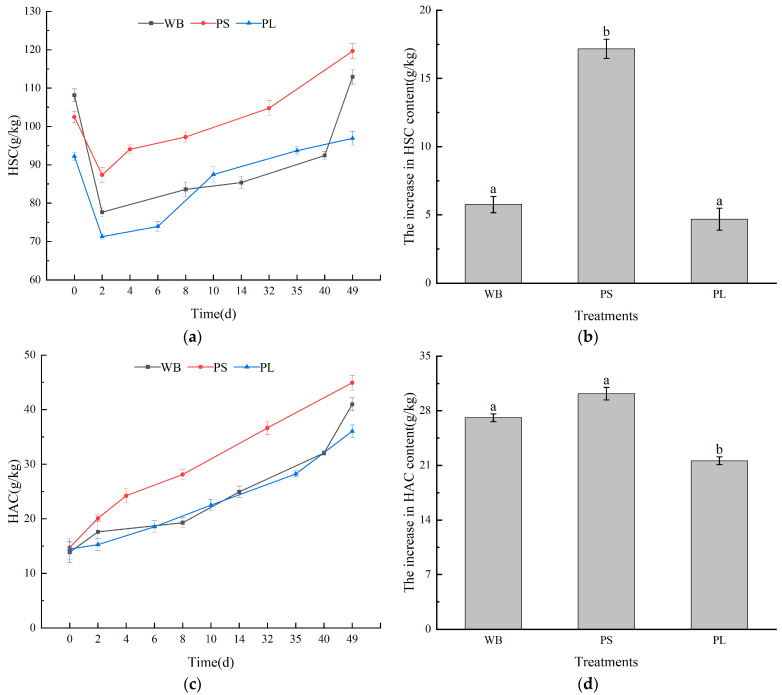

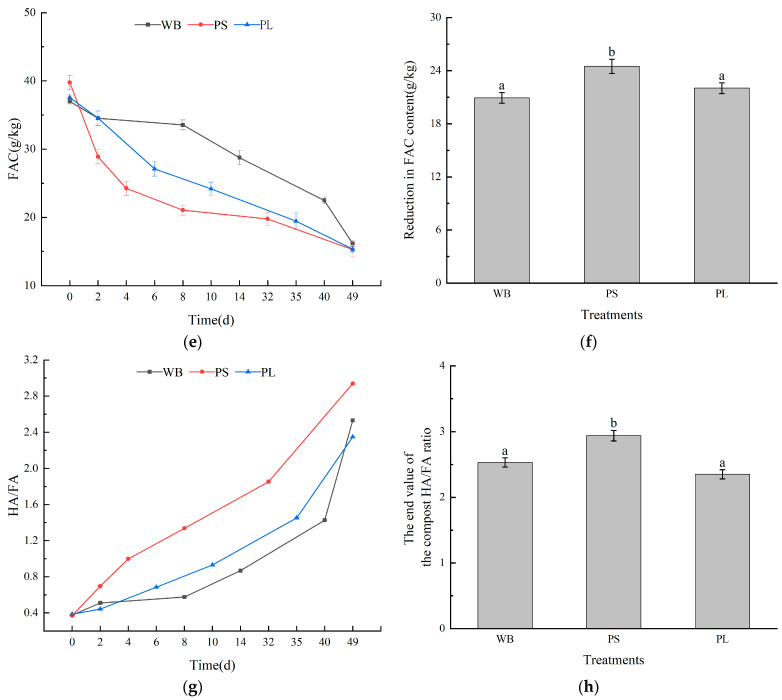
Three treatments for carbon changes in humus: (**a**) changes in the HSC content during the three composting treatments, (**b**) increases in the HSC content during the three composting treatments, (**c**) changes in the HAC content during the three composting treatments, (**d**) increases in the HAC content during the three composting treatments, (**e**) changes in the FAC content during the three composting treatments, (**f**) decreases in the FAC content during the three composting treatments, and (**c**) changes in the FAC content during the three composting treatments. (**g**) Changes in the HA/FA ratio during the composting process of the three treatments, and (**h**) the end value of the HA/FA ratio of the three treatments at the end of composting. Different letters represent significant differences between different composting treatments (*p* < 0.05).

Compost quality is principally governed by humification dynamics, with humic substances serving as terminal biomarkers of maturation [50,51]. As illustrated in Figure 7a, humus carbon (HSC) evolution across three lignocellulosic substrates exhibited biphasic kinetics characterized by initial depletion followed by progressive accumulation. This phase-dependent behavior originates from thermally labile organic fractions undergoing rapid mineralization during early thermophilic stages, whereas recalcitrant humic polymers accumulate during maturation [52]. Terminal HSC quantification revealed differential accumulation patterns: 112.92 g/kg (WB), 119.64 g/kg (PS), and 96.88 g/kg (PL), with PS demonstrating superior humification potential.

Humic acid carbon (HAC) and fulvic acid carbon (FAC) displayed divergent transformation trajectories (Figure 7c,e). Continuous HAC augmentation was observed across all treatments (66.2–67.2% increases), culminating in maximal values of 44.90 g/kg (PS) > 40.94 g/kg (WB) > 36.06 g/kg (PL). Contrastingly, FAC exhibited sustained depletion (56.3–61.6% reductions), confirming the preferential microbial conversion of labile fulvic fractions to stabilized humic polymers [53]. PS’s exceptional FAC mineralization efficiency (61.6% decrease) suggests enhanced metabolic channeling toward humification compared to other substrates.

The humification index (HA/FA ratio) provides critical insights into compost maturity [54]. All treatments surpassed the maturity threshold (HA/FA >1.9), with terminal ratios of 2.93 (PS) > 2.53 (WB) > 2.30 (PL) (Figure 7g) [55,56]. PS exhibited 87.3% ratio amplification, correlating with its accelerated maturation kinetics. Thermophilic-phase HA/FA surges (particularly in PS) likely reflect the microbial remodeling of fulvic substrates under thermal stress, where FAC serves as a redox shuttle for humification [57]. These findings align with established humification paradigms in lignocellulosic waste systems [58,59].

### 3.4. Infrared Spectrum Analysis in Composting Process

**Figure 8:**
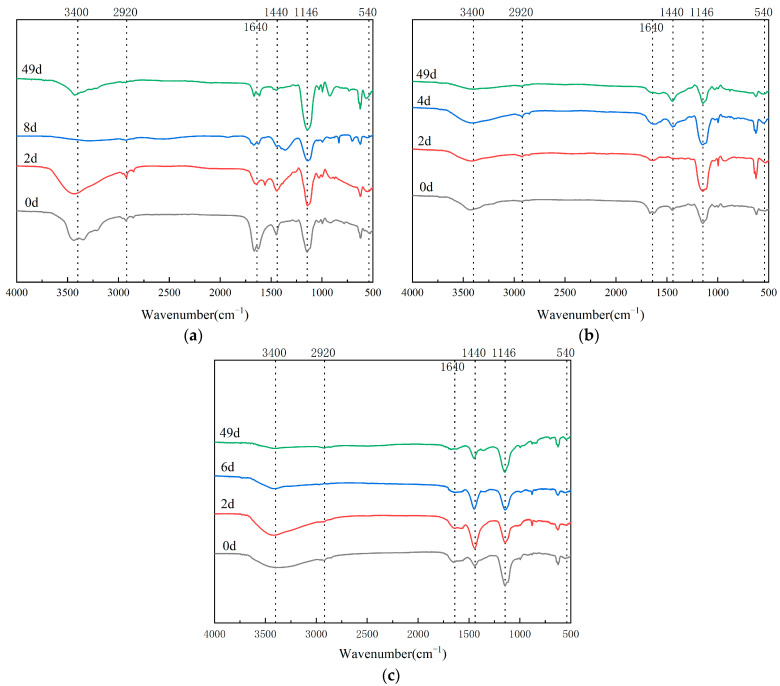
Infrared spectrum analysis of HA in the composting process of three treatments: (**a**) infrared spectrum analysis of HA in the composting process of WB treatment, (**b**) infrared spectrum analysis of HA in the composting process of PS treatment, and (**c**) infrared spectrum analysis of HA in the composting process of PL treatment.

Fourier-transform infrared spectroscopy (FTIR) provides critical insights into organic matter transformation during composting through functional group identification and spectral pattern evolution [60]. A spectral analysis of humic substances (HSs) across the three plant-derived waste composting systems revealed distinct molecular transitions (Figure 7 and Figure 8). The broad 3400 cm^−1^ absorption band, indicative of hydroxyl/carboxyl groups in carbohydrate moieties, displayed dynamic intensity variations throughout the process. Aliphatic CH stretching vibrations (2920 cm^−1^) and aromatic CC skeletal vibrations (1640 cm^−1^) demonstrated material-specific degradation patterns, particularly notable in wood-based (WB, Figure 8a) compost.

Humification progression was evidenced by the progressive enhancement of 1440 cm^−1^ signals, characteristic of carboxyl stretching in humic-like substances. PS-treated compost exhibited the most significant humification index elevation (ΔI1440 = 2.3-fold, Figure 8b), suggesting superior humus formation efficiency. Transient increases in aliphatic signatures (2920/540 cm^−1^) followed by subsequent attenuation implied microbial-mediated lipid metabolism, with the WB and PS systems showing complete aliphatic decomposition cycles (t1/2 = 15 days).

Polysaccharide dynamics (1146 cm^−1^) revealed substrate-dependent degradation patterns: the WB and PL systems displayed V-shaped intensity profiles during maturation (minimum at thermophilic phase, blue line of Figure 8a,c), contrasting with PS’s linear decline. This suggests differential microbial utilization strategies for lignocellulosic components, particularly regarding lignin accessibility in PL matrices [61].

**Figure 9:**
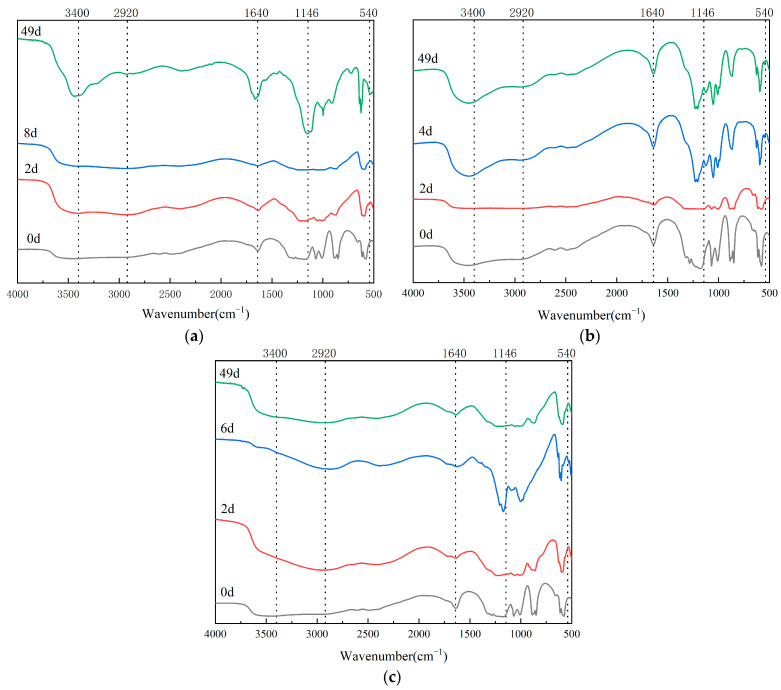
Infrared spectrum analysis of FA in the composting process of three treatments: (**a**) infrared spectrum analysis of FA in the composting process of WB treatment, (**b**) infrared spectrum analysis of FA in the composting process of PS treatment, and (**c**) infrared spectrum analysis of FA in the composting process of PL treatment.

Fulvic acid spectral evolution demonstrated inverse trends between carbohydrate-associated bands (3400 cm^−1^) and aromatic signatures (1640 cm^−1^), indicating the microbial-mediated interconversion of molecular constituents. WB treatment exhibited the most pronounced humification indicators, including the following: a 48% increase in the aromaticity index (I1640/I2920, Figure 9a); 2.1-fold polysaccharide accumulation during maturation; and a complete aliphatic-to-aromatic transition (R540 = 0.92).

These spectral transformations confirm that substrate composition governs humification pathways, with lignocellulosic wastes (WB) demonstrating superior aromatic stabilization capacity compared to herbaceous (PS) and manure-blended (PL) systems [62].

### 3.5. Compost Safety Index

Phytotoxicity evaluation through the seed germination index (GI) revealed complete substrate detoxification across treatments (GI > 80%), exceeding the critical threshold for agricultural application (GI > 50% = partial decomposition; GI > 80% = full maturation) [63]. Statistical analysis (Table 4) demonstrated significant inter-treatment variation (*p* < 0.05), with PS exhibiting superior GI performance compared to WB and PL. These findings confirm that all compost products reached optimal maturation standards while highlighting substrate-specific decomposition kinetics.

The limits of heavy metals in the international European Union organic fertilizer standard are as follows: lead (Pb)—50 mg/kg; cadmium (Cd)—3 mg/kg; chromium (Cr)—100 mg/kg; arsenic (As)—10 mg/kg; and mercury (Hg)—2.0 mg/kg [64]. In this study (Table 4), the heavy metal content of the three groups was significantly lower than the international limit value, indicating the safety of their agricultural application. Among them, the content in PS treatment was significantly lower than that in the other two treatments, which indicates its degree of maturation and stable heavy metals. The content in PL treatment was higher, which may be because the lignin content of PL is high, and the degradation of PL in compost is slow, which reduces the microbial conversion efficiency of heavy metals [65].

### 3.6. Bacterial Community Structure and Diversity During Composting

#### 3.6.1. Bacterial Community Structure

**Figure 10:**
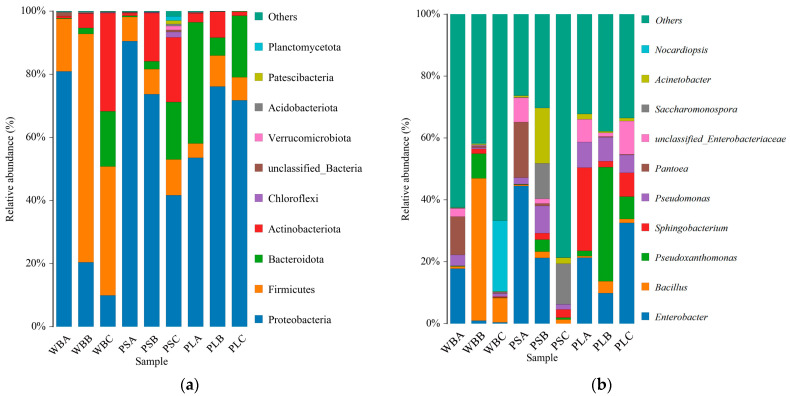
Changes in bacterial community structure: (**a**) changes in bacterial community structure at phylum level and (**b**) changes in bacterial community structure at genus level.

The community structure of bacteria in the composting process of three kinds of plant-derived waste changed significantly at the phylum level (Figure 10a). In the whole composting process, the dominant bacteria of the three piles were Proteobacteria, Firmicutes, Bacteroidota, and Actinobacteriota, and the total relative abundance reached more than 80%. Among these bacteria, the relative abundance of Proteobacteria only increased with the increase in temperature in the PL treatment. The analysis showed that at this stage, some thermophilic bacteria in Proteobacteria were the main bacteria that decompose organic matter and also play a certain role in nitrogen cycling, thus promoting composting [66]. In addition, with the increase in composting time, the relative abundance of WB and PS Proteobacteria decreased, while Firmicutes, Bacteroidota, and Actinobacteriota increased. Among them, the relative abundance of Proteobacteria in WB decreased the most, by 87.6% compared to the initial value at the decomposition stage. The relative abundance of firmicutes increased rapidly with increasing temperature, so it became the dominant phylum. Previous studies have shown that Firmicutes can effectively degrade carbohydrates [67]. Therefore, in the thermophilic period, Firmicutes accelerated the organic carbon degradation process in the WB treatment, which is consistent with the research results in Figure 4. Bacteroidota and Actinobacteriota have the ability to decompose lignin and cellulose and promote the decomposition of compost [68]. Obviously, at the initial stage of composting, organic matter such as a carbon source that is easily degraded was decomposed, and Proteobacteria decreased. During the curing stage, the proportion of refractory substances (lignin) increased, and the abundance of dominant bacteria in the WB and PS treatments changed significantly. For PL treatment with a high lignin content, the abundance of dominant bacteria was different from in the previous two treatments.

As shown in Figure 10b, the dominant bacteria genera of the bacteria in the three treatment groups at the genus level mainly include *Enterobacter*, *Bacillus*, *Pseudoxanthomonas*, and *Sphingobacterium*. With the increase in compost temperature, the relative abundance of *Enterobacter* in all three groups decreased, with the greatest decrease in PS treatment, and it decreased by 53.3% in PSB compared with PSA. *Enterobacter* abundance increased in PLC, while it significantly decreased in both WBC and PSC. This may be because the high temperature inhibited the growth of this bacterium, while the refractory lignin in poplar leaf litter was metabolized as a carbon source by Enterobacter, supporting its later growth [69].

#### 3.6.2. Bacterial Community Diversity

The ACE and Chao1 indices are used to reflect the richness of a bacterial community, while the Simpson and Shannon indices are used to reflect the diversity of a bacterial community [70]. According to the analysis of the Alpha diversity of bacteria in different plant-derived wastes and in different compost periods in Table 5, the WB and PS treatments showed significant differences in the richness of the bacterial community in the initial stage, thermophilic stage, and ripening stage, while PL treatment showed significant improvement in the richness of the bacterial community in the curing stage. Among the treatments, the PS and PL treatments had the highest richness and diversity in the ripening stage, while this was true for WB treatment in the thermophilic stage. In addition, the richness and diversity of PS compost after decomposition were higher than those of the other two types of compost.

**Figure 11:**
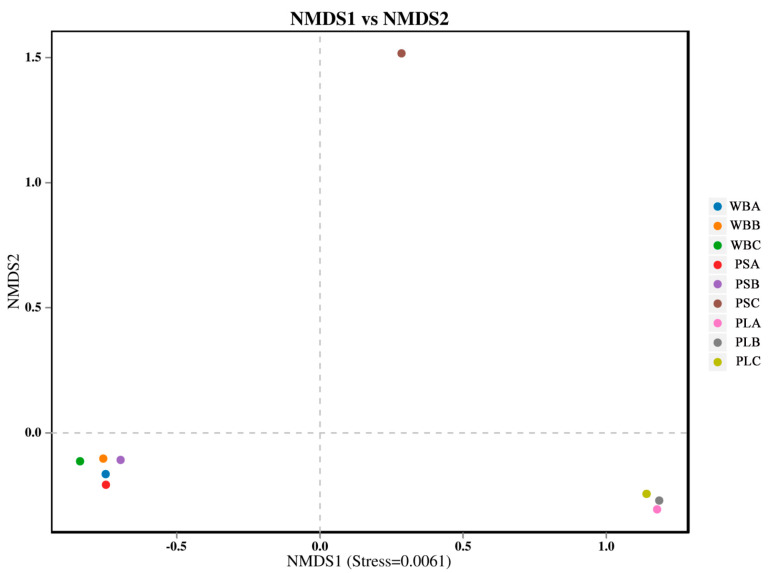
Nonmetric multidimensional scale analysis of bacterial communities (NMDS).

The Beta diversity analysis of the bacterial communities in the three groups showed that the bacterial communities in the initial and thermophilic phases of the WB and PS treatments were similar (Figure 11). However, the Beta diversity of the bacterial community in PS treatment changed significantly during the maturation stage. In addition, during the composting process, the bacterial community composition of PL treatment was significantly different from that of the WB and PS treatments, while the WB and PL treatments had no significant change. In this study, in a comparison before and after composting, PS treatment significantly increased the richness and diversity indices of soil bacterial community, followed by PL treatment. This was consistent with the results of NMDS analysis, indicating that the activity of some bacteria was inhibited in the thermophilic stage of compost. In the maturation stage, thermophilic bacteria easily decompose organic matter and produce new functional flora [71].

#### 3.6.3. Functional Analysis of Bacterial Communities

**Figure 12:**
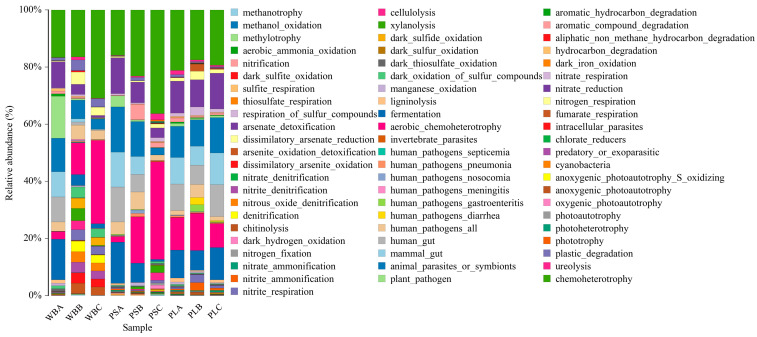
Functional analysis of bacterial communities.

As shown in Figure 12, the bacterial community function analysis results of the three groups of treatments through the FAPROTAX database showed that the main ecological functions of the WB, PS, and PL treatments in each composting stage were chemoheterotrophy, aerobic_chemoheterotrophy, and fermentation. The chemoheterotrophy and aerobic_chemoheterotrophy functions of WB and PS increased with the increase in composting time, while the two functions of PL increased first and then decreased. When the three treatments entered the thermophilic stage, the fermentation function decreased, but only the PL treatment increased when the compost matured, while the other two treatments continued to decline. In the process of composting and decomposing, the metabolism-related functions of PS treatment accounted for a relatively large proportion, followed by WB treatment, while the fermentation functions of PL treatment accounted for a relatively large proportion at the end of composting, which is similar to Jiang’s research findings [72]. In addition, microbial function prediction is a powerful tool to analyze microbial ecological mechanisms, but its essence is to rely on existing databases and infer indirectly based on statistical association [73]. In research on composting, it is necessary to analyze the changes in environmental factors.

### 3.7. Fungal Community Structure and Diversity During Composting

#### 3.7.1. Fungal Community Structure

**Figure 13:**
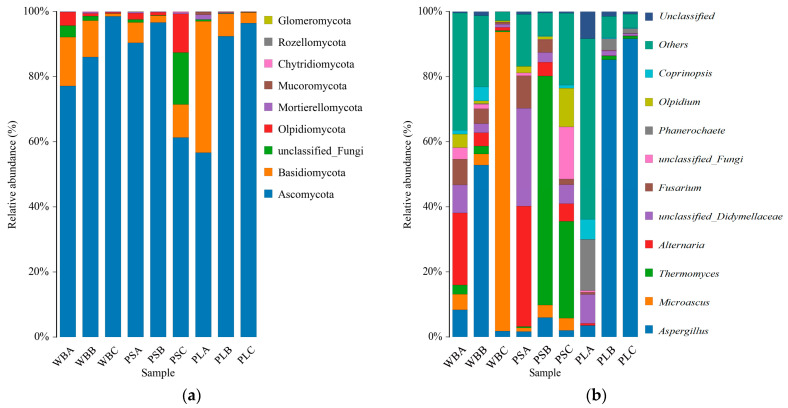
Changes in fungal community structure: (**a**) changes in fungal community structure at phylum level and (**b**) changes in fungal community structure at genus level.

According to the classification of fungi at the phylum level in the composting process of the three treatments (Figure 13a), Ascomycota and Basidiomycota were the dominant phyla in all samples, with Ascomycota being the absolute dominant group. The relative abundance of Ascomycota in the three treatments increased from 77%, 90%, and 57% to 86%, 97%, and 92%, respectively. This increase is attributed to the ability of Ascomycetes to secrete a variety of cellulose- and hemicellulose-degrading enzymes, which allows them to efficiently utilize nutrients in compost [74]. In contrast, the relative abundance of Basidiomycota decreased in all treatments, with the most significant decline observed in the PL treatment.

At the genus level, significant changes were observed in the microbial community of WB and PS during the composting process (Figure 13b). In the initial stage, *Alternaria* was the dominant genus in both WB and PS. However, in the thermophilic stage, *Aspergillus* and *Thermomyces* became dominant, respectively. The relative abundance of *Microascus* in WB increased rapidly during the curing stage, reaching 92%. The *Aspergillus* genus remained dominant in the PL treatment, with its relative abundance increasing significantly to 85% during the thermophilic stage.

#### 3.7.2. Fungal Community Diversity

According to the Alpha diversity analysis of fungi in different plant-derived wastes and in different compost periods in Table 6, the richness of the fungal community decreased significantly when the PS and PL treatments entered the thermophilic stage, and there was a significant difference compared with the initial stage, while there was no significant difference in the richness of fungi in the WB compost treatment. The diversity of the fungi community in the WB and PL treatments decreased significantly with the increase in composting time, while that in PS treatment decreased first and then increased. In addition, the fungi richness of PL treatment after composting was significantly higher than that of the other two types of compost, and the diversity index showed that the increase in the PS treatment was more significant. After aerobic composting, the diversity of WB treatment and the abundance of PS and PL decreased significantly. According to the analysis, this was because in the thermophilic stage of compost, thermophilic fungi gradually became the dominant fungal group, while other fungi that were not tolerant to high temperature were suppressed or eliminated. During compost maturation, the decomposition of organic matter tended to stabilize, and the activity of microorganisms decreased, resulting in a decrease in fungal diversity [75].

**Figure 14:**
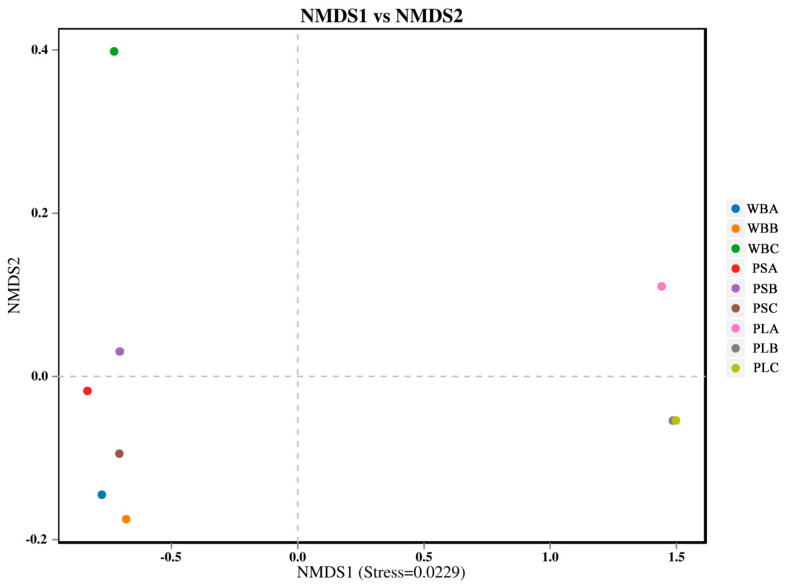
Nonmetric multidimensional scaling analysis of fungal communities (NMDS).

The Beta diversity analysis of fungal communities in the three groups showed significant changes in fungal community composition between the ripening stage and the initial stage (Figure 14). Among the groups, WB treatment had the most significant difference in fungal community composition, followed by PL treatment. In addition, the fungal community composition in the WB and PS treatments was similar in the initial and thermophilic stages, while that in PL treatment was significantly different from the other two treatments during composting and decomposition.

#### 3.7.3. Functional Analysis of Fungal Communities

**Figure 15:**
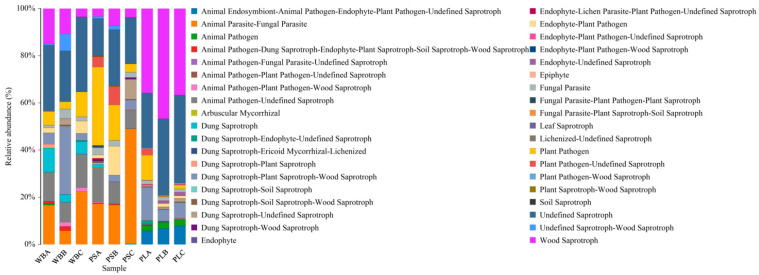
Functional analysis of fungal communities.

As shown in Figure 15, the fungal functional groups in the WB treatment were mainly concentrated in Undefined Saprotroph in the initial period and concentrated in Animal Pathogen-Undefined Saprotroph after entering the thermophilic period. In the final ripening stage, groups were concentrated in Undefined Saprotroph and Animal Parasite-Fungal Parasite. In the initial stage of PS treatment, groups were mainly concentrated in Plant Pathogen and Animal Parasite-Fungal Parasite. During composting, the functional fungi of Animal Parasite-Fungal Parasite increased rapidly and significantly. After composting and decomposing, the proportion of plant pathogens in the main functional flora of PS treatment was greatly reduced. This indicates that the use of aerobic composting technology can reduce the potential impact of peanut straw as an organic fertilizer on plant diseases [76]. In the whole composting process in the PL treatment, the functional group of fungi was mainly concentrated in Wood Saprotroph and Undefined Saprotroph, and the related functional fungi of Wood Saprotroph increased first and then decreased with the increase in composting time. This indicates that PL treatment can better degrade lignin into humus [43].

### 3.8. Correlation Between Microbial Community Structure and Nutrient Indicators in Compost

**Figure 16:**
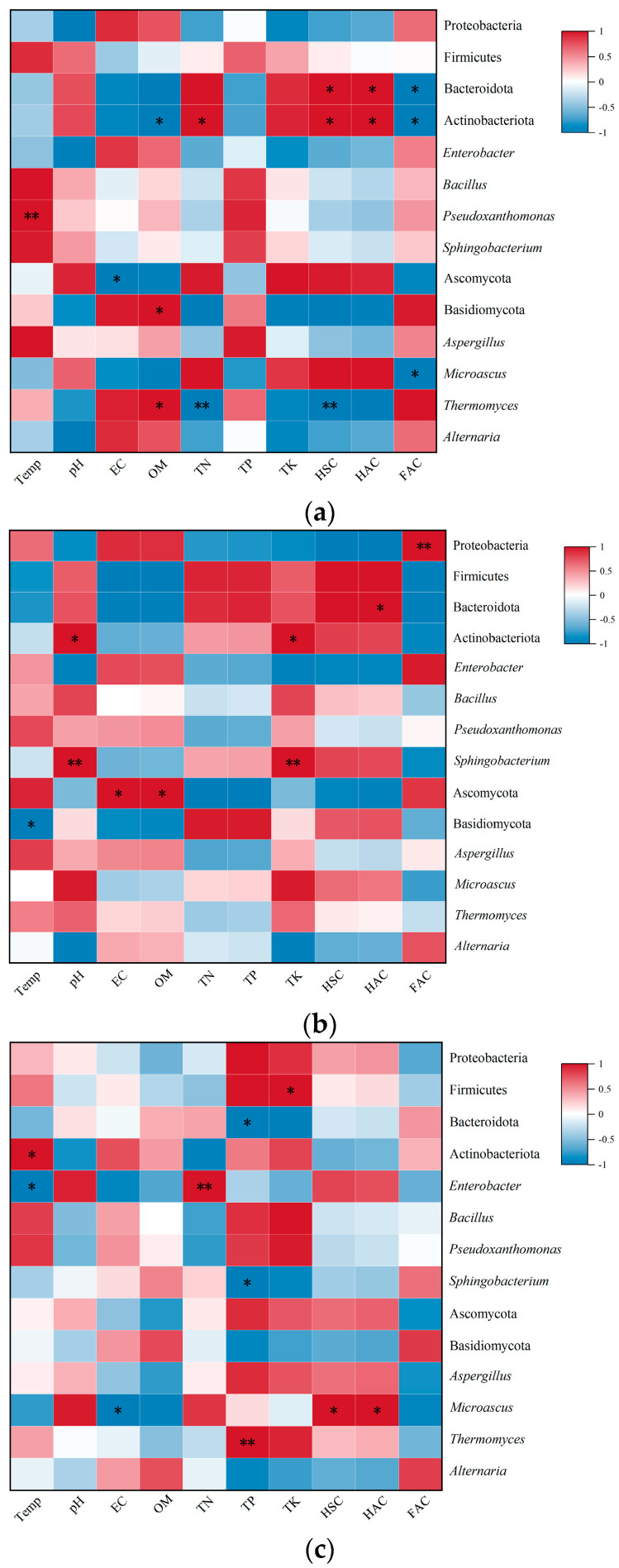
Correlation between core microbial communities and environmental factors: (**a**) WB treatment, (**b**) PS treatment, and (**c**) PL treatment. * and ** indicate significant (*p* < 0.05) and extremely significant (*p* < 0.01) correlation, respectively.

Figure 13 illustrates the multivariate relationships between microbial consortia and physicochemical parameters during plant-derived waste composting [77]. Microbial dynamics exhibited treatment-specific associations with compost maturation markers. In WB treatment systems (Figure 16a), Bacteroidota and Actinobacteriota demonstrated strong positive correlations with humic substance components (HSC, HAC) but inverse relationships with fulvic acid content (FAC). Notably, *Actinobacteriota* abundance displayed contrasting responses to organic matter (OM; negative) and total nitrogen (TN; positive), while *Pseudoxanthomonas* thermotolerance showed temperature-dependent proliferation during thermophilic phases, suggesting its dual regulatory role in carbon/nitrogen cycling [78]. Fungal analyses revealed Ascomycota’s inverse correlation with electrical conductivity (EC), whereas Basidiomycota and *Thermomyces* exhibited OM-dependent growth patterns. The thermophilic-phase reduction in *Thermomyces* coincided with the maturation-phase accumulation of TN and HSC in WB systems [79].

The PS treatment compost (Figure 16b) demonstrated divergent bacterial responses, with Proteobacteria showing exceptional FAC affinity (initial relative abundance >90%), potentially driving fulvic acid biotransformation. Bacteroidota selectively associated with humic acid accumulation, while Actinobacterial members correlated with pH and total potassium (TK) dynamics. The thermophilic *Sphingobacterium* population appeared crucial for acidic environment modulation and potassium mobilization, aligning with algal waste composting mechanisms [64]. Fungal communities exhibited temperature-dependent Basidiomycota suppression and EC/SOC-responsive Ascomycota activation.

PL treatment systems (Figure 16c) displayed minimal bacterial–environment correlations, though Enterobacter’s biphasic dynamics (thermophilic decline/mesophilic resurgence) mirrored TN accumulation patterns during maturation. Fungal analyses identified *Thermomyces*-mediated phosphorus cycling through thermophilic-phase TP correlation. These findings collectively suggest nutrient-specific microbial regulation, with WB systems governed by thermal–TN–HSC interactions, PS by pH-TK-FAC dynamics, and PL by TN-TP cycling [80,81].

### 3.9. Structural Equation Model of Humification Degree of Compost

**Figure 17:**
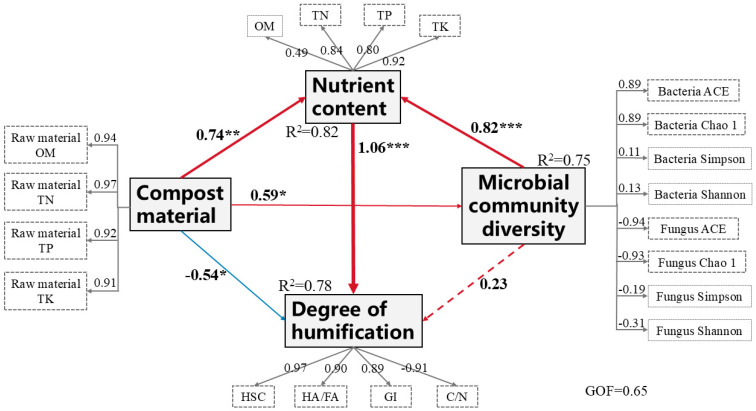
Structural equation model of the effects of compost material, nutrient content, and microbial community structure on the humification degree of compost. * means *p* < 0.05, ** means *p* < 0.01, and *** means *p* < 0.001. The red line indicates the positive path, and the blue line indicates the negative path. The width of the line indicates the degree of influence. The values next to the lines are the path coefficients, and the dashed lines indicate insignificant effects. R^2^ represents the proportion of the explained variance. Microbial community diversity was expressed using the α-diversity index. The degree of humification was indicated by humification indicators (HSC content, HA/FA, GI value, and C/N).

The Partial Least Squares Path Modeling (PLS-PM) approach enables visual representation of dynamic intervariable relationships [82]. The goodness-of-fit (GOF) metric evaluates overall model performance in explaining data variability, with higher values denoting superior explanatory capacity [83]. The constructed structural equation model revealed intricate associations among compost substrates, nutritional parameters, and microbial diversity in governing humification processes, demonstrating variable impacts (positive, neutral, or negative) on compost maturation [84]. As illustrated in Figure 17, the model effectively elucidated these relationships, achieving a GOF value of 0.65, indicative of satisfactory model fit. Variance explanation rates reached 82% for nutrient content, 75% for microbial diversity, and 78% for humification degree.

Nutrient content exhibited the strongest positive influence on humification (β = 1.06), with total potassium (TK) displaying the highest factor loading, followed by total nitrogen (TN) and total phosphorus (TP), all reaching statistical significance. Compost substrates significantly enhanced nutrient levels (β = 0.74) and microbial diversity (β = 0.59) but inversely affected humification (β = −0.54). Among substrate components, TN showed predominant factor loading, while organic matter (OM), TP, and TK also contributed significantly.

Microbial diversity, quantified through α-diversity indices (ACE and Chao1), showed no direct effect on humification (β = 0.23, ns) but substantially promoted nutrient accumulation (β = 0.82). Bacterial and fungal communities exerted contrasting effects, with bacterial richness positively correlated and fungal abundance negatively associated with nutrient dynamics. Humification indicators revealed distinct patterns: the humic substance carbon (HSC) content demonstrated the highest factor loading, positively linked to the humic/fulvic acid ratio (HA/FA) and germination index (GI), yet inversely related to C/N ratio. These findings corroborate previous evidence that reduced C/N ratios enhance compost humification.

Therefore, nutrient content was the most significant direct factor affecting the humification degree of different compost materials. The change in microbial community diversity in different compost materials was a key driver of the humification degree. This showed that the microbial community structure greatly affected nutrient circulation and accumulation during composting, thus promoting the degree of compost maturation. The application effect of three kinds of plant-derived waste compost as organic fertilizer still needs to be further studied.

In general, the degree of humification is ultimately determined by the combined action of the chemical composition, physical structure, and microbial selection of compost raw materials. For the material with a high-nitrogen source (WB), its nutrient content is higher, the particles are fine, and it is easy to decompose, but the loss rate of carbon and nitrogen is higher in the composting process. For materials with a high-carbon source (PL), the initial carbon level is too high, which will inhibit microbial activity and reduce the humification degree. Materials (PS) with a higher lignin content in their own structure need to be crushed before composting, which is more conducive to their compost maturation. According to the results of this study, high-quality organic fertilizer can be achieved.

## 4. Conclusions

The composting trials demonstrated the effective decomposition and stabilization of diverse plant-derived wastes, meeting established aerobic composting criteria. In the process of compost decomposition, PS treatment first entered the thermophilic stage, WB treatment reached the highest temperature, and high temperature was long-lasting. The final maturation parameters showed that the pH value of the three treatments was alkaline, and the EC value was less than 4 mS/cm. PS treatment compost displayed optimal maturation indicators, including heavy metal contents that were significantly lower than the average standard limits, a 94.8% germination index, and 17.4% organic matter mineralization. Critical nutrient transformations were observed: total nitrogen increased by 84.9%, phosphorus by 52.0%, potassium by 60.7%, and the humic substance content rose 16.7%, culminating in a favorable humic/fulvic acid ratio of 2.93. Fourier-transform infrared spectroscopy confirmed enhanced humification in the WB and PS treatments, with PS-derived humic acids demonstrating higher aromaticity and structural stability. Microbial community analysis revealed distinct dynamics: PS treatment fostered significantly greater bacterial diversity, whereas PL compost showed elevated fungal richness. These microbial shifts correlated strongly with humification progression. In general, after 49 days of composting and decomposing, the peanut straw waste pile had a higher degree of decomposition. Changes in the microbial community diversity of the three plant-derived waste composts were the key drivers of the degree of humification.

The results of this study can be applied in real-world practice. The composting method used in this study can effectively convert materials into mature fertilizer, significantly improving soil health and soil fertility. In terms of the environment, the economic cost of composting treatment is reduced. At the same time, it reduces the environmental pressure on landfill waste and helps to reduce greenhouse gas emissions. And it can be used as a reference for the composting of other plant-derived waste. Overall, this study improves the resource utilization efficiency of plant-derived waste compost and provides theoretical support for sustainable agriculture and circular economy practices.

## Figures and Tables

**Table 1:** Design of compost treatment group.

Test Treatment	Organic Matter (g/kg)	Total Nitrogen (g/kg)	Compost C/N	Moisture Content (%)	Raw Material (kg)	Urea (g)
WB	748.06	29.12	30/1	65–70%	10	140
PS	681.38	15.64	30/1	65–70%	10	130
PL	609.17	4.01	30/1	65–70%	10	110

**Table 2:** Sampling time of the compost from different plant-derived wastes.

Sample Collection	WB	PS	PL
Initial Stage	2d (9.26)	2d (9.26)	2d (9.26)
Peak Thermophilic Stage	8d (10.2)	4d (9.28)	6d (9.30)
Thermophilic Stage	14d (10.10)	8d (10.2)	10d (10.4)
Cooling Stage	40d (11.3)	32d (10.26)	35d (10.29)
Curing Stage	49d (11.12)	49d (11.12)	49d (11.12)

**Table 3:** Sample sequencing table.

Test Grouping	Sampling Time	Sampling Day	Number of Samples
WB	Initial period	26 September 2023	WBA
	Thermophilic period	2 October 2023	WBB
	Curing period	12 November 2023	WBC
PS	Initial period	26 September 2023	PSA
	Thermophilic period	28 September 2023	PSB
	Curing period	12 November 2023	PSC
PL	Initial period	26 September 2023	PLA
	Thermophilic period	30 September 2023	PLB
	Curing period	12 November 2023	PLC

**Table 4:** Seed germination index in three compost treatments.

Samples	Germination Index (%)	Pb (mg/kg)	Cd (mg/kg)	Cr (mg/kg)	As (mg/kg)	Hg (mg/kg)
WB	87.56 a	18.2 ± 2.3 a	1.8 ± 0.3 a	45.7 ± 5.2 a	4.2 ± 0.6 a	0.7 ± 0.1 ab
PS	94.79 b	9.5 ± 1.1 b	0.6 ± 0.1 b	28.3 ± 3.8 b	1.9 ± 0.2 b	0.2 ± 0.05 b
PL	80.32 c	24.6 ± 3.0 c	2.5 ± 0.4 c	62.4 ± 6.5 c	6.8 ± 0.9 c	1.1 ± 0.2 a

Note: different letters represent different treatments with significant differences (*p* < 0.05).

**Table 5:** Statistics of Alpha diversity index of bacteria in samples.

Samples	ACE Index	Chao1 Index	Simpson Index	Shannon Index
WBA	636.65 ± 9.38 Aa	636.50 ± 7.23 Aa	0.94 ± 0.05 Aa	5.36 ± 0.12 Aa
WBB	808.35 ± 13.75 Ba	810.00 ± 10.04 Ba	0.95 ± 0.07 Aa	6.15 ± 0.13 Ba
WBC	773.51 ± 15.99 Ca	774.33 ± 6.33 Ca	0.93 ± 0.09 Aa	5.47 ± 0.23 Aa
PSA	925.34 ± 10.88 Ab	925.66 ± 5.67 Ab	0.95 ± 0.07 Aa	5.63 ± 0.03 Aa
PSB	825.15 ± 6.42 Ba	825.00 ± 8.82 Ba	0.94 ± 0.06 Aa	5.83 ± 0.03 Aa
PSC	1273.84 ± 11.65 Cb	1274.20 ± 9.57 Cb	0.97 ± 0.07 Ba	7.95 ± 0.33 Bb
PLA	300.19 ± 15.13 Ac	300.00 ± 15.07 Aa	0.97 ± 0.03 Aa	6.55 ± 0.18 Ab
PLB	299.60 ± 15.58 Ab	300.50 ± 9.27 Ab	0.87 ± 0.05 Bb	5.18 ± 0.35 Ba
PLC	562.00 ± 12.82 Bc	562.00 ± 7.06 Bc	0.97 ± 0.07 Aa	6.61 ± 0.43 Ac

Note: different capital letters represent significant differences in the same pile in different composting periods; different lowercase letters represent significant differences between different piles in the same composting period (*p* < 0.05).

**Table 6:** Statistics of Alpha diversity index of fungi in samples.

Samples	ACE Index	Chao1 Index	Simpson Index	Shannon Index
WBA	62.55 ± 6.38 Aa	62.66 ± 13.67 Aa	0.92 ± 0.04 Aa	4.74 ± 0.27 Aa
WBB	69.06 ± 10.67 Aa	70.75 ± 15.77 Aa	0.82 ± 0.04 Aa	3.93 ± 0.31 Aa
WBC	63.93 ± 10.44 Aa	66.25 ± 10.82 Aa	0.22 ± 0.04 Ba	1.04 ± 0.35 Ba
PSA	117.62 ± 8.74 Ab	118.14 ± 17.29 Ab	0.76 ± 0.06 Ab	3.26 ± 0.84 Ab
PSB	67.28 ± 7.28 Ba	69.20 ± 11.35 Ba	0.50 ± 0.04 Bb	2.19 ± 0.65 Bb
PSC	63.40 ± 12.28 Ba	73.00 ± 9.15 Ba	0.86 ± 0.08 Ab	3.94 ± 0.26 Ab
PLA	309.56 ± 13.16 Ac	309.50 ± 16.22 Ac	0.95 ± 0.05 Aa	5.81 ± 0.51 Ac
PLB	198.16 ± 9.88 Bb	203.11 ± 9.11 Bb	0.54 ± 0.02 Bb	2.24 ± 0.19 Bb
PLC	225.10 ± 11.13 Bb	225.27 ± 14.27 Bb	0.50 ± 0.07 Bc	1.88 ± 0.47 Ba

Note: different capital letters represent significant differences in the same pile in different composting periods; different lowercase letters represent significant differences between different piles in the same composting period (*p* < 0.05).

## Data Availability

Data are contained within the article.
